# Regional inequality and vaccine uptake: a multilevel analysis of the 2007 Welfare Monitoring Survey in Malawi

**DOI:** 10.1186/1471-2458-12-1075

**Published:** 2012-12-13

**Authors:** Dawit Shawel Abebe, Vibeke Oestreich Nielsen, Jon Erik Finnvold

**Affiliations:** 1 NOVA – Norwegian Social Research, Munthesgate, Oslo, Norway; 2 Statistics Norway, Kongensgate, Oslo, Norway

**Keywords:** Vaccination coverage, Regional inequality, Social inequality, Malawi, Survey, Multilevel analysis

## Abstract

**Background:**

A significant part of childhood mortality can be prevented given the existence of a well functioning health care system that can deliver vaccines to children during their first year of life. This study assesses immunization differentials between regions in Malawi, and attempts to relate regional disparities in immunization to factors on individual, household and village level.

**Method:**

We used data from the 2007 Welfare Monitoring Survey which includes 18 251 children ages 10–60 months. Multilevel logistic regression models were applied for data analysis.

**Results:**

Major differences in full vaccine coverage (children receiving all of the 9 recommended vaccines) were documented between the 27 official regions, called districts, of Malawi. The vaccine coverage among regions varied from 2% to 74% when all children 10 – 60 months old were included. Vaccine coverage was significantly higher for women that had their delivery attended by a midwife/nurse, or gave birth at a hospital or maternity clinic. Regions with a high percentage of deliveries attended by health personnel were also characterized by a higher coverage. Characteristics of health care utilization on the individual level could in part account for the observed regional variations in coverage.

Several factors related to socio-demographic characteristics of individuals and households were significantly correlated with coverage (child’s age, illiteracy, income, water and sanitary conditions), implying a lower coverage among the most vulnerable parts of the population. However, these factors could only to a minor extent account for the regional variation in coverage.

**Conclusions:**

The persistent regional inequalities suggest that the low immunization coverage in Malawi is less likely to be a result of geographical clustering of social groups with difficult level-of living conditions. Although the mean vaccine coverage in Malawi is low, some regions have succeeded in reaching a relatively high proportion of their children. The relative success of some regions implies that there is a substantial potential for political intervention to improve vaccine coverage. One important negative implication of regional inequality is the presence of clusters with under-vaccinated children, leading to an increased vulnerability during outbreaks of vaccine-preventable diseases.

## Background

It is estimated that about 8.8 million children younger than 5 years died in 2008, and that about half of these deaths occurred in sub-Saharan Africa [1]. According to the WHO, at least 63% of these deaths could have been prevented by vaccination [2]. Health interventions like vaccination programmes should obviously be implemented in places where they are most needed. WHO has advocated the Reaching Every District (RED) approach as a way to improve immunization performance [3]. The RED approach includes several operational components designed to improve uptake in every district, including supportive supervision and on-site training. The RED approach also encourages countries to utilise statistics on the uptake of vaccines to analyse the distribution of un-immunized children.

The context of this study is Malawi, a low income country with a population of about 13 million of which 84,7 percent live in rural areas [4]. Vaccination of children is done by local health facilities, either at clinics or at mobile outreach personnel including Health Surveillance Assistants (HSAs), who cover every village in Malawi [5,6]. The vaccination is free of charge. However, there is a substantial shortage of health personnel in Malawi and one person may therefore be responsible for a large number of people. The information and follow up may therefore be insufficient, especially in rural areas, and vaccinations may either not be given on time or not at all. In addition, significant stock-outs of BCG and DPT vaccines at the central level were reported in 2007 [7].

The purpose of this paper is to identify regions in Malawi in which uptake of vaccines is significantly below or above the national average, and to suggest explanations for the regional variations in vaccine uptake displayed in the 2007 Welfare Monitoring Survey.

Vaccine uptake may markedly differ between regions for several possible reasons. The key question is why these variations exist. Vaccinations are delivered in local contexts, by professionals and semi-professionals supported by authorities at higher regional levels in an organizational hierarchy. Material or infrastructural resources such as the availability, accessibility and quality of local health care facilities may play a significant part in explaining regional variations. Based on a Nigerian survey, Antai and collaborators found that families in regions with a relatively high proportion of births delivered in hospitals had higher vaccination rates [8]. A similar finding is also reported in another cross-sectional study from Nigeria [9]. It follows that variations in vaccine uptake can be conceived of as indicators of performance in health care. These are socially constructed features of the local environment that provide opportunities for families.

Travelling distance to health care facilities have been found to be strongly correlated with vaccination uptake [10]. Regional inequalities in health worker density may also exist, as evidence from Tanzania has suggested [11].

Other community level processes that are relevant for vaccine uptake presuppose some form of social interaction. Knowledge and the diffusion of knowledge about vaccination opportunities is one case in point. It is documented that knowledge and discussions about vaccination improve uptake [12]. The relative proportion of literate adults in local communities may also have a general positive effect on the presence of health knowledge in local communities [13]. Literacy not only improves the situation for those that have an education, but might also have an effect on the uptake of vaccines for those that are not literate but live in regions with a relatively high proportion of literacy.

A considerable bulk of health research has traced the connections between vaccine uptake and socio-cultural characteristics of individuals and households [14]. Several studies, in particular from South Asia, have documented severe gender inequalities and strong preferences toward male offspring due to cultural or traditional customs [15]. This phenomenon has not been observed in sub-Sahara. However, one study from rural Malawi has reported a lower mortality among 1–2 year old rural male children [16]. Other factors at the individual and household level, such as parental education, literacy and occupation may be important, as well as indicators of cultural factors including belief and trust in health professionals. Uptake tends to be lower in households with lower socioeconomic status and in households characterized by general poor living conditions such as poor housing, sub-standard sanitary or fresh-water facilities [8,12,17-21]. These factors may affect individual’s demand for vaccinations as well as their propensity to accept the offer of vaccination. In addition, people are to varying degrees embedded in the localities they reside. People with larger social networks are more likely to receive information about vaccines. The relationship between social capital and health is well known [22], but the consequences for uptake of immunization is less well understood.

According to *compositional explanations*, regional variations in vaccine uptake occur because individuals or households with low vaccine uptake tend to be geographically clustered. Accordingly, we attempt to empirically assess the relative importance of compositional explanations by including factors such as educational attainments, illiteracy and level-of-living indicators of the household (income, water and toilet facilities) in the empirical analysis. Furthermore, we include information about individual’s relations with, and access to, local health facilities. In addition to individual-level information based on the 2007 welfare monitoring survey, we also include regional-level variables that describe variations health care and level-of living conditions.

## Methods

### Data: the 2007 welfare monitoring survey

The analysis in this article is based on data from the 2007 Welfare Monitoring Survey (WMS) in Malawi which was one of eight modules in the National Census of Agriculture and Livestock (NACAL) collected by the National Statistical Office (NSO). The survey took place autumn 2007 over a period of about two weeks and data on 21 090 children below 5 years are included in the survey. In this study, we restricted our analysis among those children ages 10–60 months (N=18 251), i.e. the sample population.

Since the main purpose of the NACAL was related to agriculture, the first sampling step included only households with land and animals. For the WMS module, additional landless households were selected so that the probability of being selected for the WMS was the same for households with and without land. The sampling of enumeration areas included both urban and rural areas, further documentation can be fond on the Malawi National Statistical Office webpage [23].

The household head was asked questions about all family members, which makes it possible to decide whether there are other children in the household than the household head’s own children, whether any of the children are orphans and whether the parent or guardian has an education. Questions about the vaccination status of all the children in the sample population of the household were asked. Variables on housing and health conditions, poverty indicators and distance to important infrastructure are also included. Since the child and household information is connected to the NACAL village module, vaccination coverage may also be tracked down to region and village level and to information of family structure and ethnicity.

In addition to the WMS, regional data on delivery attended by health care personnel were added to the dataset. We also included a regional variable reporting the percentage of the population living in a permanent dwelling as a general indicator of regional variations in level of living conditions. The regional data is documented in a publication issued by the National statistical office in Malawi [24].

### Outcome: measuring vaccination coverage

All households involved in the survey were asked questions for each child less than 5 years on whether it received measles, DPT (three doses), Polio (4 doses) and BCG vaccinations. The response was based both on information from vaccination cards (88%) and/or from mother/guardians recollection (12%). It is specified whether a vaccination card is shown, but no information exists on when the vaccination was given. This makes it impossible to decide whether the child got the vaccination at the recommended age. Without vaccination cards, recall bias might be an issue. We decided to include families without a health card. The decision was informed by previous assessments of the quality of child immunization coverage estimates in population based surveys, that found no major systematic weakness in recall data [25]. Full immunization coverage included children that have received all of the nine vaccinations. The outcome variable is a dichotomy, identifying children with full vaccination coverage and children with less than full coverage.

### Individual, household and health care characteristics based on the WMS (first level variables)

Characteristics related to child include gender and year of birth. Two variables indentify mother’s school attendance and literacy status (able to read and write). *Mother’s marital status* consist 6 different categories, never married, married monogamous, married polygamous, divorced, separated and widowed. The study also includes a poverty estimate that divides the households into five quintiles based on the estimated annual household consumption per capita in the WMS. The estimate has been calculated using a poverty model [26] and is independent of the variables used to explain the variation in vaccination coverage in this study.

Health care experiences include two variables related to the birth of the child: where the child was delivered (five categories: hospital/maternity clinic, health clinic, health centre, health post, at home, other), and who assisted with the delivery of the child (four categories: doctor/clinical officer, midwife/nurse, trained traditional birth attendant (t.b.a), self). A variable describing walking distances to the nearest health care facility was also included.

### Regional (second level) variables

Regional level variables comprise information about the percentage of deliveries assisted by trained personnel, and percentage of the population living in permanent dwellings. The sources of these variables are not from the 2007 WMS, but based on the 2008 Malawi Population and Housing Census, documented in Table 1.

**Table 1:** Regional variations in vaccine coverage, deliveries attended by trained personnel and permanent dwelling. Percent. (N)

	**Vaccine coverage. Percent fully vaccinated**^**1**^	**Number of observations**^**1**^	**Percent living in permanent dwelling – 2007**^**2**^	**Percent delivered by trained personell 2007 – 2008**^**3**^
Total	33	17868	41	42
Likoma	83	18	-	-
Nsanje	74	470	31	61
Salima	64	534	47	51
Ntcheu	60	616	70	50
Kasungu	57	775	52	25
Chikwawa	56	550	54	39
Mwanza	55	479	62	60
Chiradzulu	52	568	61	49
Ntchisi	46	571	68	43
Dedza	39	812	58	32
Dowa	38	595	56	38
Phalombe	38	626	63	56
Lilongwe/Lilongwe city	37	1558	36	43
Thyolo	36	712	78	40
Mzimba/Mzuzu city	30	1037	60	47
Machinga	29	604	83	60
Zomba/Zomba city	26	1151	60	41
Karonga	25	658	49	34
Blantyre/Blantyre city	21	794	36	30
Mulanje	14	490	77	44
Nkhotakota	14	585	33	41
Balaka	14	546	59	42
Mangochi	11	1014	49	34
Mchinji	9	685	60	42
Chitipa	6	483	68	53
Nkhata bay	6	475	52	37
Rumphi	2	462	41	100

^1^Source: 2007 welfare monitoring survey. Missing observations for vaccine coverage was 2.1% (N=383).

^2^Source: Ministry of health and population. Table 3.9, Malawi Statistical yearbook 2009. Reproductive health care by district july 2007 – june 2008: http://www.nso.malawi.net/images/stories/data_on_line/general/yearbook/Yearbook_2009/Chapter%203.xls.

^3^Source: Table 5.3, p.52 in The 2008 Malawi Population and Housing Main report: http://www.nso.malawi.net/images/stories/data_on_line/demography/census_2008/Main%20Report/ThematicReports/Spatial%20Distribution%20and%20Urbanisation.pdf.

### Statistical analysis

The data were analyzed using two-level logistic regression models (mixed models), where individual-level factors considered as lower-level predictors and the regional-level factors were considered as higher-level predictors. We estimated both fixed and random effects; the first explains the variation of vaccine coverage at the individual-level and the later explains the variation of vaccine coverage at the regional-level. First, we developed random intercept models, explaining the variability of full vaccine coverage at the individual-level (fixed effects) and also explaining the regional-level variation by random intercepts, but the regression slopes are assumed fixed. We adopted the forward selection strategy where factors were added step by step at each model through evaluating their effects by p-values and fit index (log likelihood). In order to best fit the models or in the absence of improved log likelihood values, predictors with a p-value greater than 0.2 were dropped from analysis. Secondly, we developed random-slope modes that may explain variability at the regional-level. For the simplicity of model estimation, each predictor was added one by one in the random component of models. And also, nominal variables (e.g. where the child was delivered and who assisted the delivery of child) were dictomized. Effects of predictors were evaluated by coefficients for slopes and changes in log likelihood as compared to the random-intercept model. In general, we conducted a step-by-step generation of a basic model using a model contrasting approach illustrated by log likelihood values. P-values less than or equal to 0.05 was considered as the level of significance. Maximum likelihood estimates were applied. Statistical analysis was carried out using Stata SE/11 for Windows.

### Ethical considerations

The authors have used data from the Malawi Welfare Monitoring Survey (WMS). The survey was carried out by the National Statistical Office (NSO) in Malawi which followed rules for confidentiality and anonymity. Statistics Norway, through the institutional cooperation with NSO, has given professional input to several stages of the data collection, but neither of the authors have been involved in this work. Statistics Norway has received official permission from NSO to use the dataset for vaccination research. A report on general findings from the WMS is publicly available.

## Results

### Regional diversity in vaccine coverage

**Figure 1:**
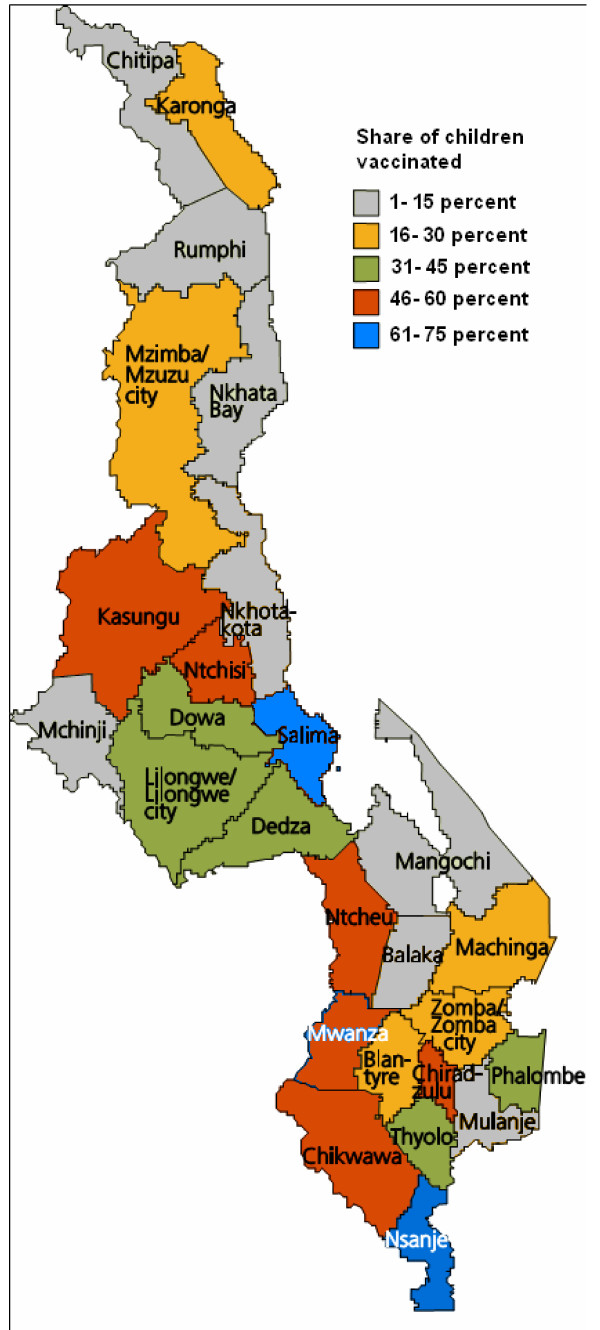
Variations in vaccine coverage, regions in Malawi.

Malawi consists of 27 administrative regions. The variations in vaccination coverage are displayed in Table 1, which also documented regional variations in deliveries assisted by trained personnel, and percentage of the population living in permanent dwellings. If we exclude Likoma, a small group of islands in Lake Malawi and with only 21 observations in the WMS, the highest ranked region was Nsanje, with coverage of 74%. Seven regions had coverage over 50%. The four regions with the lowest ranking had less than 10%vaccine coverage. Although there is no clear pattern to where the regions with high and low vaccine coverage are situated, many of the districts in the Northern Region are among those with low vaccination rates (see Figure 1). A closer inspection of the data revealed that the regional variation displayed in Table 1 were robust to changes in age groups and the exclusion of children without vaccination cards. We will therefore include all children ages 10–60 months in our study, since the number of observations is highest in this case.

Although the full vaccination rate is very low in some regions, the rates are substantially higher for many of the single vaccines (Link to Additional file 1: Table S1 detailing coverage for each separate vaccine).

In Table 2, descriptive statistics with information on vaccination uptake are presented for all the variables included in the analysis. Variations related to gender, family typology and education appear to be small or non-existent. A minor gradient seems to exist regarding household consumption per capita, in which the poorer households have lower coverage. Children born in 2006 naturally have markedly lower coverage since many of the children have not yet filled one year and have therefore not yet received all vaccinations.

**Table 2:** Vaccination coverage. Percent. (N)

**Individual**		**N**
*Child male or female*		
Male	33	8035
Female	33	8047
*Childs year of birth*		
2002	36	753
2003	33	4251
2004	36	4515
2005	33	4637
2006	28	3712
*Mothers marital status*		
Never married	33	404
Married, monogamous	33	12780
Married, polygamous	30	1062
Divorced	32	675
Separated	33	759
Widowed	32	410
*Mother ever attended school?*		
Yes	32	11756
No	35	4297
*Can mother read and write a simple sentence in any language?*		
Yes	32	9903
No	33	6240
**Household**		
*Estimated annual household consumption pr.capita*		
Lowest quintile	29	4762
2	31	4012
3	35	3444
4	35	2839
Highest quintile	36	1661
*Water source*		
Unsafe	29	3603
Safe	34	14233
*Toilet facility*		
Improved pit latrine	31	1017
Pit latrine	33	15111
None	31	1677
**Health care**		
*Where was the child delivered?*		
Hospital/maternity clinic	36	6086
Health clinic	35	1993
Health centre	33	3769
Health post	24	410
At home	30	4875
Other	21	621
*Who assisted the delivery of the child?*		
Doctor/clinical officer	36	1968
Midwife/nurse	35	9805
Trained t.b.a.	30	3780
Other	26	1561
Self	26	578
*Minutes to walk to the nearest health clinic or hospital*		
0-14	32	863
15-29	32	1630
30-44	32	2288
45-49	33	2580
60+	33	19390
*Trust to hospital staffs*		
All	32	7791
Most	33	4580
Some	33	3434
Few	31	1507
None	41	442

Missing values was between 2.1% and 2.8% for the single items included in table.

Variables describing the circumstances around the delivery of the child seem to have a modest impact on coverage. In general, coverage is higher for mothers that used more specialized health care facilities during birth, and for mothers that had qualified health personnel present during birth. More surprisingly, walking distances to health care facilities do not seem to have an impact on vaccine coverage.

In Table 3, we presented a series of random intercept models which included predictors for the full vaccine coverage at the individual-level and the regional-level. The final fitted models were only presented in Table 3. Details about the analysis are available from authors upon request. In Model 1, child’s age and gender were included as predictors for the full vaccine coverage. Children born in 2006 had significantly lower odds to be fully vaccinated compared to those born in 2002. Model 2 included maternal factors, suggesting that mothers who could not read and write a simple sentence had a significantly lower probability to fully vaccinate their child. As to predictors related to the household income and conditions (Model 3), the model indicated that children in households with a higher annual income (those in the 3^rd^ and 4^th^ quintiles) had a significantly higher likelihood to be fully vaccinated as compared to those children in the lowest quintile group. Similarly, households with unsafe water source and without toilet facilities were significant predictors for not being fully vaccinated. Since gender (p= 0.769) and marital status (p=0.844) had no significant effects, we dropped it from further analysis.

**Table 3:** Multilevel logistic mixed models results for predictors on the full vaccine coverage of children in Malawi

**Factors**	**Model 1**	**Model 2**	**Model 3**	**Model 4**	**Model 5**
	**OR(95% CI)**	**OR(95% CI)**	**OR(95% CI)**	**OR(95% CI)**	**OR(95% CI)**
**Fixed effects:**					
**Child factors**					
*Gender*					
Male	1.0	-	-	-	-
Female	1.01(0.94-1.09)	-	-	-	-
*Childs year of birth*					
2002	1.0	1.0	1.0	1.0	1.0
2003	0.87(0.73-1.06)	0.88(0.73-1.07)	0.87(0.72-1.06)	0.83(0.67-1.03)	0.83(0.67-1.03)
2004	0.98(0.81-1.17)	0.98(0.81-1.19)	0.99(0.82-1.21)	0.98(0.79-1.22)	0.98(0.80-1.22)
2005	0.83(0.69-1.00)	0.83(0.69-1.01)	0.82(0.68-1.01)	0.81(0.65-1.00)	0.81(0.66-1.00)
2006	0.64(0.53-0.78)***	0.66(0.54-0.87)***	0.66(0.54-0.80)***	0.64(0.52-0.79)***	0.64(0.52-0.79)***
**Maternal factors**					
*Mothers marital status*					
Single	-	1.0	-	-	-
Married	-	0.94(0.75-1.19)	-	-	-
Divorced	-	0.99(0.74-1.33)	-	-	-
Separated	-	0.89(0.67-1.18)	-	-	-
Widowed	-	0.95(0.68-1.32)	-	-	-
*Mother ever attended school?*					
Yes	-	1.0	1.0	1.0	1.0
No	-	1.12(0.99-1.25)	1.10(0.98-1.25)	1.09(0.95-1.25)	1.09(0.96-1.25)
*Can mother read and write a simple sentence in any language?*					
Yes	-	1.0	1.0	1.0	1.0
No	-	0.81(0.72-0.89)***	0.88(0.78-0.99)*	0.88(0.77-0.99)*	0.88(0.77-0.99)*
**House-hold factors**					
*Estimated annual household consumption per capital*					
Lowest quintile	-	-	1.0	1.0	1.0
2	-	-	1.05(0.94-1.17)	1.05(0.94-1.19)	1.06(0.94-1.19)
3	-	-	1.19(1.06-1.34)**	1.12(0.99-1.28)	1.12(0.99-1.26)
4	-	-	1.19(1.06-1.36)**	1.13(0.99-1.24)	1.13(0.99-1.29)
Highest quintile	-	-	1.15(0.99-1.34)	1.05(0.90-1.23)	1.05(0.90-1.23)
*Water source*					
Safe	-	-	1.0	1.0	1.0
Unsafe	-	-	0.78(0.71-0.85)***	0.81(0.73-0.91)***	0.81(0.73-0.90)***
*Toilet facility*					
Improved pit latrine	-	-	1.0	1.0	1.0
Pit Latrine	-	-	0.85(0.71-1.01)	0.92(0.77-1.11)	0.92(0.77-1.08)
None	-	-	0.61(0.49-0.75)**	0.73(0.58-0.92)**	0.73(0.58-0.92)**
**Health care**					
*Where was the child delivered?*					
Hospital/maternity clinic	-	-		1.0	1.0
Health clinic	-	-		0.94(0.82-1.07)	0.94(0.82-1.07)
Health centre	-	-		0.75(0.67-0.84)***	0.75(0.67-0.88)***
Health post	-	-		0.62(0.45-0.85)**	0.62(0.45-0.85)**
At home	-	-		0.92(0.72-1.17)	0.92(0.72-1.18)
*Who assisted the delivery of the child?*					
Doctor/clinical officer	-	-		1.0	1.0
Midwife/nurse	-	-		1.32(1.16-1.50)***	1.31(1.15-1.50)***
Trained t.b.a.	-	-		1.04(0.80-1.35)	1.04(0.80-1.35)
Self	-	-		0.66(0.47-0.94)*	0.66(0.47-0.94)*
*Minutes to walk to the nearest health clinic or hospital*					
0-30	-	-	-	1.0	1.0
31-59	-	-	-	1.09(0.95-1.18)	1.09(0.95-1.18)
60+	-	-	-	0.99(0.88-1.13)	0.99(0.87-1.13)
*Trust to hospital staffs*					
All	-	-	-	1.0	1.0
Most	-	-	-	1.06(0.96-1.18)	1.07(0.96-1.18)
Some	-	-	-	1.05(0.94-1.18)	1.05(0.94-1.18)
Few				0.96(0.82-1.11)	0.96(0.83-1.12)
None	-	-	-	1.00(0.77-1.31)	1.00(0.77-1.31)
**Regional Factors**					
*Delivery attended by Health personnel (%)*	-	-	-	-	0.98(0.95-1.01)
*Permanent dwelling (%)*	-	-	-	-	1.01(0.97-1.04)
*Log likelihood*	−8877.4	−8688.1	−8175.6	−7129.3	−7122.6

*p < 0.05, **p < 0.01, ***p < 0.001; OR= adjusted odds ratio; CI= confidence interval.

In Model 4, factors related to health care were examined by including the place of delivery, who assisted the delivery of child, access (time to walk) to nearest health institutions and the level of trust to hospital staffs. Children born in health centre and health post had significantly lower odds to be fully vaccinated compared to those who were born in maternity clinics. Moreover, there was a higher chance of being fully vaccinated if the delivery was assisted by midwifes or nurses than if the delivery was assisted by doctors or clinical officers. In contrast, the delivery assisted by mothers themselves had a significantly lower probability to be fully vaccinated. Factors related to access (time to walk) to nearest health institutions and the level of trust to hospital staff had no significant effects, but retained in the further analyses due to improved log likelihood values.

In Model 5, the regional-level factors, proportion of delivery attended by health personnel and proportion of permanent dwelling were added as continuous variables and neither of them had a significant effect. In general, predictors in the final model (Model 5) also resulted in considerable improvements of the log likelihood, reflecting the superior fit of this model compared to the earlier models – factors in the fitted model have a significant contribution in explaining the vaccine coverage among the study population. Furthermore, we developed the caterpillar plot (Additional file 2: Figure S1) using random intercept estimates and standard errors from the Model 5. As shown in Additional file 2: Figure S1, the residual plots of 26 regions, one for each region, support the between regions variability for the vaccination coverage, and also indicate a necessity for the random slope model.

In the above mentioned models, we only included random intercepts, so that our next models were primarily developed to explain the variation of full vaccine coverage at the regional-level by a random slope. For this purpose, we continued to fit a random slope in the final fitted model in Table 3 (Model 5), and presented the random component and fit index in Table 4. These include the individual-level factors such as household income, maternal education, where the child was delivered and who assisted the delivery of child. Except for self-attended delivery, those who born in health centre/post and home, and maternal education; the random-slope models for other predictors have considerable improvements in the log likelihood values, reflecting the superior fit of these random-slope models in predicting the variability of full-vaccine coverage across regions compared to the nested random-intercept model. Moreover, most parameter estimates are much larger than the corresponding standard errors, showing the significance (p<0.05) effects of random slopes: in regions with larger coefficients for household income, delivery attended by doctors and midwife, and child delivery at hospitals/maternity clinic slope, these factors have a larger impact on full-vaccine coverage, and vice versa.

**Table 4:** Random-slope models for explaining the regional-level variation of full vaccine coverage

**Random effects**	No random slope*	**Household income**	**Delivery assisted by**			**Where was the child delivered?**			**Maternal education**
			**Doctor**	**Midwife**	**Self**	**Hospital /maternity clinic**	**Health center/post**	**Home**	
Intercept=S.d.(S.E)	1.22(0.17)	1.30(0.19)	1.26(0.18)	1.30(0.19)	1.122(0.17)	1.23(0.18)	1.25(0.18)	1.18(0.17)	0.88(0.15)
Slope=S.d.(S.E)	-	0.15(0.03)	0.75(0.15)	0.33(0.07)	0.46(0.22)	0.47(0.09)	0.47(0.09)	0.39(0.10)	0.39(0.08)
Log Likelihood	−7122.6	−7101.5	−6839.0	−6858.7	−7121.3	−7099.8	−7716.2	−7728.2	−7719.9

*Derived from Model 5; S.d.= standard deviation; S.E= standard error.

## Discussion

In this study, we have documented a considerable variation in vaccine coverage between the 27 districts in Malawi. A number of household characteristics related to the living conditions (low income, unsafe water source and lack of toilet facilities) were associated with lower vaccine coverage. These findings is consistent with results from previous DHS-investigations in Malawi (1992, 2000 and 2004), reporting that the most vulnerable social groups have less access to public health services [27]. To some extent, maternal illiteracy also affected vaccine coverage. However, household and maternal characteristics could only to a minor extent account for the regional variation on coverage.

At the individual level, vaccine coverage was significantly related to several indicators of health care utilization. As found in a number of previous studies [8,28,29], mothers that had deliveries attended by nurses or midwives, or gave birth at a hospital or maternity clinic, were more likely to have fully vaccinated children. Moreover, even though these factors were measured at the individual level, they explained a considerable variation in the vaccination coverage at the regional level.

We suggest two possible interpretations for the aforementioned finding. First, the population in different areas is more or less inclined to utilize health care facilities. Health care could be locally available, but people may well differ in their conceptions and knowledge about the possible benefits of the health services. Second, it may simply reflect lack of available health care resources. Lack of qualified health personnel and substandard quality of health care are common in many east African countries [11,30]. If deliveries are relatively often assisted by health care personnel in one region, it is likely that the overall health care facilities, including outreach teams, are relatively available and well functioning. The relative merit of these two interpretations is difficult to assess based on the available information in the survey. However no correlation was found between vaccine coverage and trust toward hospital staff. The implication of this finding can be that a supply-side explanation is more plausible. Further investigation is highly recommended.

Vaccine coverage was 30% for children born at home, compared to 35% for children born in hospital or maternity clinic (Table 2). In the multivariate models children born at home did not have a significantly lower chance of being vaccinated. Home delivery in Malawi is mainly practiced by households with relatively difficult level-of living conditions [27]. Several of the variables related to level of living conditions (income, water source, toilet facility) were associated with vaccine coverage. When adjusting for these factors, home delivery was not significant.

The analysis also concluded that vaccination rates were higher for children born with the presence of midwife/nurse compared to those born with the assistance of a doctor or clinical officer. This finding emphasize the importance of the nursing profession in vaccine coverage.

Long travelling distances did not seem to have an effect on coverage. This finding suggest that a “friction of distance” is not present in Malawi in the same way as reported in other sub-Saharan countries [29,31] as well as in other parts of the world . One possible explanation is that the outreach teams in Malawi may function in a uniform way throughout the country.

Unlike the experience from countries in Asia [15], and in line with previous findings in Malawi and other sub-Saharan countries [28,31], no differences in coverage were found between boys and girls. As for the child’s year of birth, vaccine coverage was significantly lower for children born in 2006. This may suggest a lack of adherence to principles of timeliness, as incomplete and incorrect vaccinations may be included in the measures of vaccination coverage [31]. Another option is that the results reflect a downward trend in vaccine coverage, also reported in a previous study from Malawi [28].

## Conclusions

Compositional explanations for regional variations in vaccine coverage are only helpful in a modest degree in the case of Malawi. To a large extent, the regional variation in vaccine coverage is a contextual phenomenon. Given the considerable variation reported between regions, a policy that focus on area rather than social groups are more likely to result in improvements in coverage. Future research, both quantitative and qualitative design, is highly needed, particularly to understand why some regions succeed in achieving high and appropriate vaccine uptake, while others are less successful in this respect. To improve our understanding of regional inequalities, more relevant contextual or regional data is needed. Information about possible shortages of supply of vaccines, and statistics on availability and turnover of health personnel might be useful candidates.

## Competing interests

The authors declare that they have no competing interests.

## Authors’ contributions

JEF was the principal investigator. JEF and DSA analysed the data, interpreted the results and drafted the manuscript. VON prepared the data material, and participated partly in data analysis. All authors participated in the write-up, contributed to the interpretation of the study results and approved the final version of the manuscript submitted for publication.

## Pre-publication history

The pre-publication history for this paper can be accessed here:

http://www.biomedcentral.com/1471-2458/12/1075/prepub

## Supplementary Material

Additional file 1 ** Table S1.** Vaccine coverage for 10-60 month old children among regions in Malawi. Click here for file

Additional file 2 ** Figure S1.** Caterpillar plot for the regional variability. Click here for file
