# Supplementary material for: Regional inequality and vaccine uptake: a multilevel analysis of the 2007 Welfare Monitoring Survey in Malawi
Source: BMC Public Health. 2012 Dec 13;12:1075. doi: 10.1186/1471-2458-12-1075 (PMC3543726; doi:10.1186/1471-2458-12-1075)
Supplement: Additional file 1 — Table S1. Vaccine coverage for 10-60 month old children among regions in Malawi. [file 1471-2458-12-1075-S1.doc]

Additional Table A. Vaccine coverage for 10-60 month old children among regions in Malawi

|  |  | **Measles** | **BCG** | **DPT1** | **DPT2** | **DPT3** | **Polio 0** | **Polio 1** | **Polio 2** | **Polio 3** | **ALL 9** | **Observations*** |
| --- | --- | --- | --- | --- | --- | --- | --- | --- | --- | --- | --- | --- |
| Northern region | |  |  |  |  |  |  |  |  |  |  |  |
|  | Chitipa | 73 | 28 | 30 | 29 | 75 | 33 | 31 | 29 | 73 | 6 | 499 |
|  | Karonga | 77 | 49 | 53 | 49 | 78 | 50 | 55 | 49 | 78 | 25 | 663 |
|  | Rumphi | 71 | 25 | 28 | 28 | 72 | 27 | 27 | 28 | 72 | 2 | 467 |
|  | Nkhata ba | 73 | 31 | 29 | 27 | 70 | 31 | 29 | 26 | 72 | 6 | 484 |
|  | Likoma | 100 | 90 | 89 | 89 | 100 | 84 | 89 | 89 | 100 | 83 | 20 |
|  | Mzimba | 79 | 48 | 52 | 50 | 80 | 49 | 51 | 50 | 81 | 28 | 801 |
|  | Mzuzu cit | 86 | 54 | 55 | 52 | 86 | 55 | 55 | 49 | 83 | 36 | 276 |
| Central region | |  |  |  |  |  |  |  |  |  |  |  |
|  | Kasungu | 89 | 89 | 90 | 87 | 87 | 68 | 84 | 83 | 85 | 57 | 790 |
|  | Ntchisi | 87 | 80 | 80 | 76 | 75 | 63 | 80 | 78 | 75 | 46 | 584 |
|  | Dowa | 86 | 73 | 76 | 72 | 85 | 54 | 73 | 69 | 83 | 39 | 617 |
|  | Nkhota ko | 79 | 55 | 53 | 51 | 76 | 38 | 56 | 52 | 77 | 14 | 596 |
|  | Salima | 91 | 90 | 89 | 86 | 86 | 70 | 90 | 90 | 89 | 64 | 545 |
|  | Dedza | 89 | 83 | 83 | 77 | 82 | 53 | 79 | 75 | 79 | 40 | 822 |
|  | Ntcheu | 93 | 93 | 91 | 88 | 87 | 70 | 89 | 87 | 86 | 60 | 621 |
|  | Lilongwe rural | 83 | 62 | 64 | 61 | 80 | 54 | 63 | 61 | 80 | 39 | 1.328 |
|  | Lilongwe urban | 84 | 48 | 47 | 46 | 87 | 44 | 48 | 46 | 86 | 28 | 277 |
|  | Mchinji | 74 | 39 | 39 | 35 | 75 | 38 | 41 | 35 | 75 | 9 | 706 |
| Southern region | |  |  |  |  |  |  |  |  |  |  |  |
|  | Balaka | 80 | 40 | 41 | 38 | 79 | 35 | 40 | 38 | 77 | 14 | 555 |
|  | Mangochi | 76 | 38 | 39 | 36 | 73 | 40 | 40 | 36 | 72 | 11 | 1.031 |
|  | Machinga | 82 | 53 | 53 | 51 | 84 | 49 | 52 | 51 | 84 | 29 | 621 |
|  | Zomba rural | 85 | 42 | 43 | 41 | 86 | 44 | 44 | 41 | 86 | 22 | 881 |
|  | Zomba urban | 87 | 52 | 52 | 51 | 87 | 52 | 52 | 51 | 87 | 38 | 296 |
|  | Chirazulu | 90 | 86 | 86 | 86 | 91 | 60 | 86 | 86 | 92 | 52 | 575 |
|  | Blantyre rural | 81 | 45 | 46 | 48 | 82 | 43 | 48 | 47 | 82 | 24 | 579 |
|  | Blantyre urban | 82 | 36 | 41 | 39 | 82 | 34 | 40 | 40 | 83 | 15 | 234 |
|  | Thyolo | 87 | 62 | 61 | 60 | 88 | 52 | 60 | 60 | 88 | 36 | 719 |
|  | Mulanje | 81 | 51 | 52 | 51 | 83 | 28 | 51 | 50 | 83 | 14 | 511 |
|  | Phalombe | 88 | 71 | 72 | 71 | 85 | 52 | 74 | 74 | 87 | 38 | 632 |
|  | Mwanza | 95 | 98 | 98 | 96 | 92 | 63 | 95 | 95 | 94 | 55 | 484 |
|  | Chikwawa | 90 | 89 | 87 | 82 | 82 | 66 | 84 | 80 | 82 | 56 | 555 |
|  | Nsanje | 93 | 96 | 96 | 95 | 94 | 84 | 93 | 91 | 89 | 74 | 482 |
|  |  |  |  |  |  |  |  |  |  |  |  |  |
| **Malawi total** | | 84 | 61 | 61 | 59 | 82 | 50 | 61 | 59 | 82 | 33 | 18.251 |
| * The number of actual observations is a little less and varies for the different vaccines because of missing values. | | | | | | | | | | | | |
