# Supplementary figures and images for: Regional inequality and vaccine uptake: a multilevel analysis of the 2007 Welfare Monitoring Survey in Malawi
Source: BMC Public Health. 2012 Dec 13;12:1075. doi: 10.1186/1471-2458-12-1075 (PMC3543726; doi:10.1186/1471-2458-12-1075)

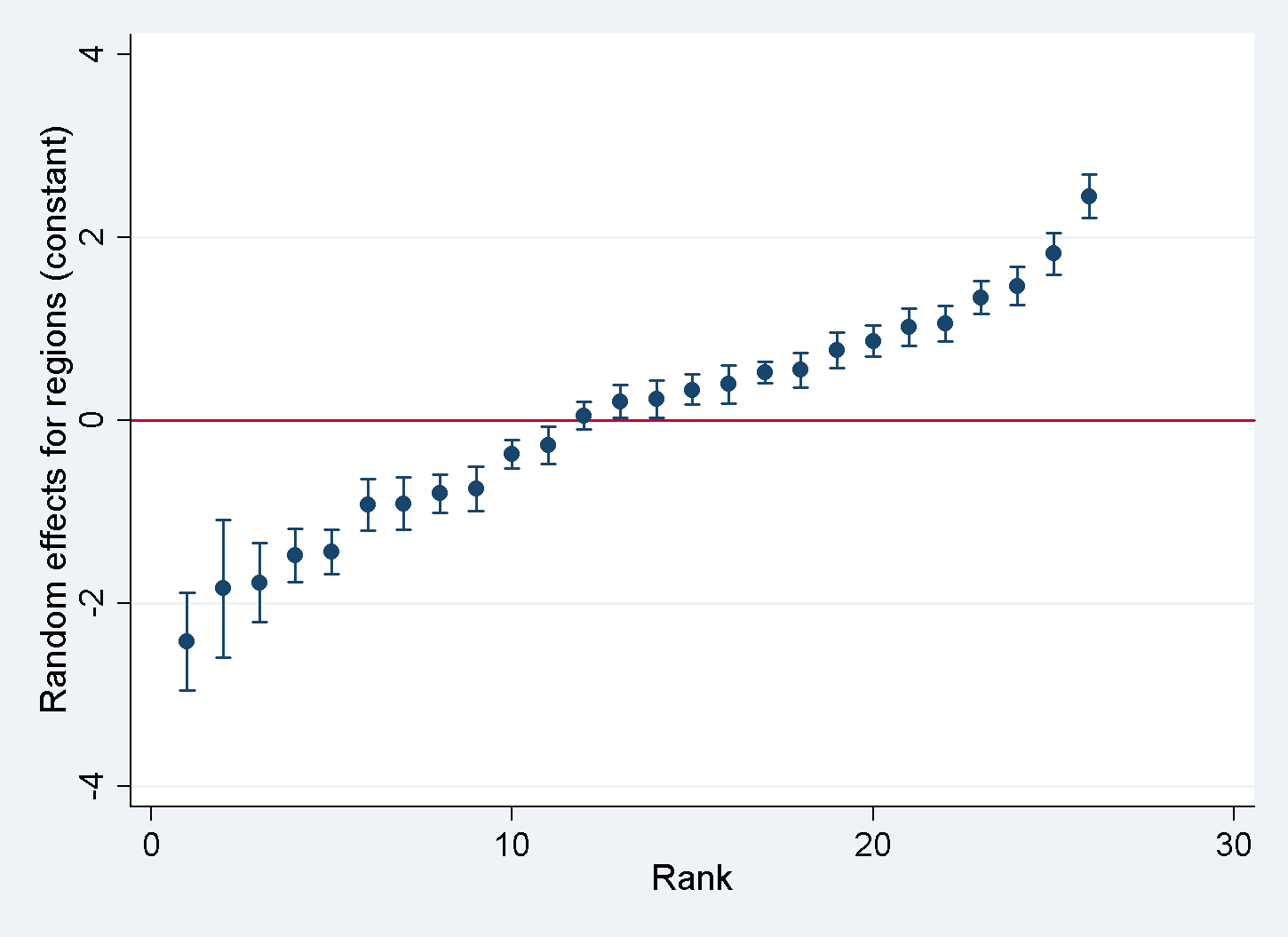

Supplement: Additional file 2 — Figure S1. Caterpillar plot for the regional variability. [file 1471-2458-12-1075-S2.tiff]
